# Supplementary material for: Selective remodelling of the adipose niche in obesity and weight loss
Source: Nature. 2025 Jul 9;644(8077):769–79. doi: 10.1038/s41586-025-09233-2 (PMC12367556; doi:10.1038/s41586-025-09233-2)
Supplement: Supplementary file 1 — Supplementary Information [file 41586_2025_9233_MOESM1_ESM.pdf]

nature portfolio

https://doi.org/10.1038/s41586-025-09233-2

Supplementary information

Selective remodelling of the adipose niche in obesity and weight loss

In the format provided by the authors and unedited

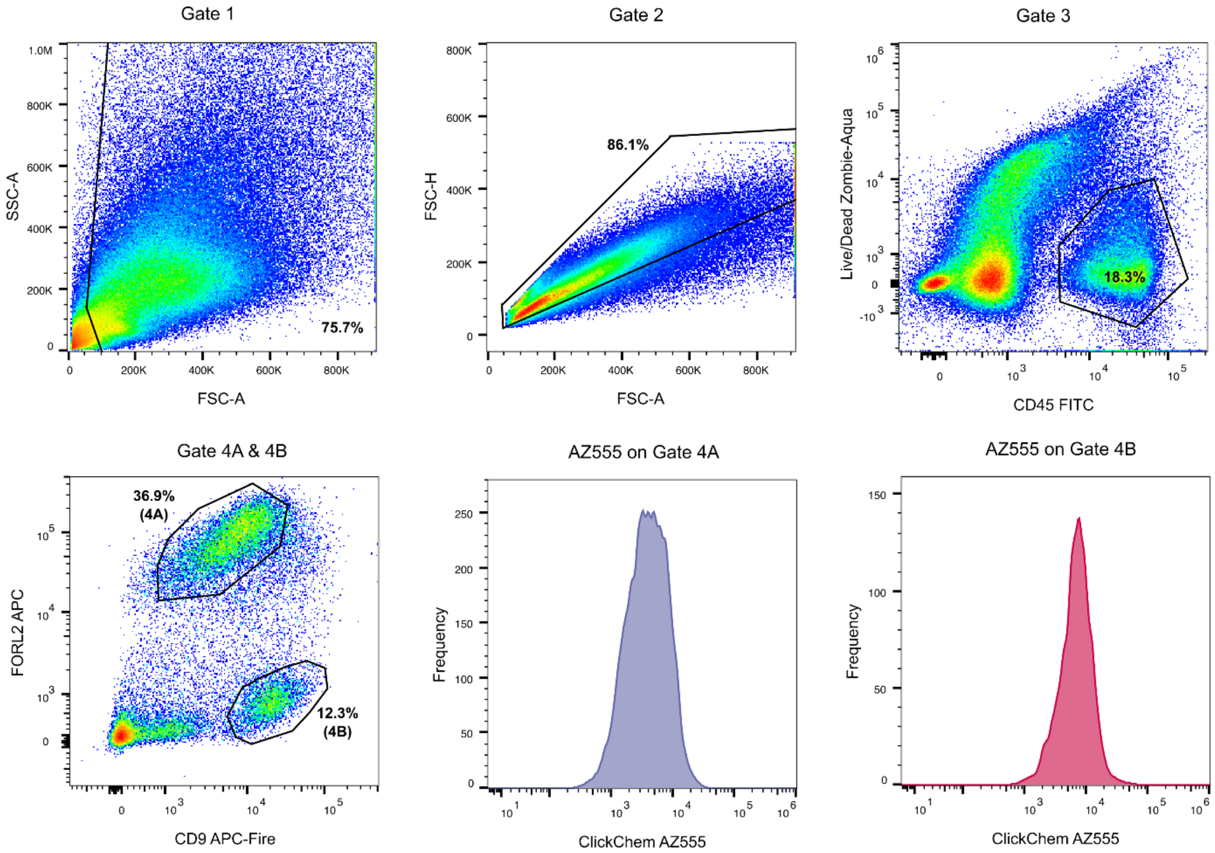

Supplementary Fig. 1: LAM and TRM gating for SCENITH-based bioenergetic studies.

Gate 1: All cells (FSC-A and SSC-A). Gate 2: Single cells (FSC-A and FSC-H). Gate 3: Live immune cells (CD45-hi, Zombie-Aqua-lo). Gate 4A: TRM cells (FORL2-hi, CD9-lo). Gate 4B: LAM cells (FORL2-lo, CD9-hi). AZ555: Click Chemistry histogram of AZ555 for respective Gates 4A and 4B. Fluorochrome and autofluorescence signatures were identified in unstained aliquots of each sample using the "AF Finder" software feature, were used to unmix the signals in fully stained samples with the built-in WLSM algorithm.
