# Supplementary material for: Selective remodelling of the adipose niche in obesity and weight loss
Source: Nature. 2025 Jul 9;644(8077):769–79. doi: 10.1038/s41586-025-09233-2 (PMC12367556; doi:10.1038/s41586-025-09233-2)
Supplement: Supplementary file 2 — Reporting Summary [file 41586_2025_9233_MOESM2_ESM.pdf]

nature portfolio

Corresponding author(s): William Scott

Last updated by author(s): Apr 29, 2025

Reporting Summary

Nature Portfolio wishes to improve the reproducibility of the work that we publish. This form provides structure for consistency and transparency in reporting. For further information on Nature Portfolio policies, see our Editorial Policies and the Editorial Policy Checklist.

Statistics

For all statistical analyses, confirm that the following items are present in the figure legend, table legend, main text, or Methods section.

n/a	Confirmed	
☐	☒	The exact sample size (*n*) for each experimental group/condition, given as a discrete number and unit of measurement
☐	☒	A statement on whether measurements were taken from distinct samples or whether the same sample was measured repeatedly
☐	☒	The statistical test(s) used AND whether they are one- or two-sided<br>*Only common tests should be described solely by name; describe more complex techniques in the Methods section.*
☐	☒	A description of all covariates tested
☐	☒	A description of any assumptions or corrections, such as tests of normality and adjustment for multiple comparisons
☐	☒	A full description of the statistical parameters including central tendency (e.g. means) or other basic estimates (e.g. regression coefficient) AND variation (e.g. standard deviation) or associated estimates of uncertainty (e.g. confidence intervals)
☐	☒	For null hypothesis testing, the test statistic (e.g. *F*, *t*, *r*) with confidence intervals, effect sizes, degrees of freedom and *P* value noted<br>*Give P values as exact values whenever suitable.*
☒	☐	For Bayesian analysis, information on the choice of priors and Markov chain Monte Carlo settings
☐	☒	For hierarchical and complex designs, identification of the appropriate level for tests and full reporting of outcomes
☐	☒	Estimates of effect sizes (e.g. Cohen's *d*, Pearson's *r*), indicating how they were calculated

Our web collection on statistics for biologists contains articles on many of the points above.

Software and code

Policy information about availability of computer code

Data collection	Raw single nucleus sequencing data: CellRanger (v5.0.1), bcl2fastq (v2.20.0)<br>Raw spatial transcriptomic data: Xenium Analyser (v1.7.1.0)<br>Human GWAS data: SHAPEIT (v2.r900), IMPUTE2 (v2.3.2)
Data analysis	Data preprocessing and quality control: CellBender (v0.2.0), Seurat (v4.3.0), SeuratObject (v4.1.3), Scanpy(v1.9.3)<br>Sample assignment from genotpye: Vireo (v0.5.6), cellsnp-lite (v1.2.2)<br>Integration: Harmonypy (v0.0.6), BBKNN (v1.6.0)<br>Spatial Segmentation: FiJi (v1.54f)<br>Exploratory data analysis: Nebula (v1.4.1), CellChat (v1.6.1), Compass (v0.9.10.2), pySenic (v0.12.1), MiloR (v1.10.0), ENVI (v0.3.6), ClusterProfiler (v4.2.2)<br>Data analysis pipelines used in this work can be obtained from: https://github.com/WRScottImperial/WAT_single_cell_analysis_Nature_2024

For manuscripts utilizing custom algorithms or software that are central to the research but not yet described in published literature, software must be made available to editors and reviewers. We strongly encourage code deposition in a community repository (e.g. GitHub). See the Nature Portfolio guidelines for submitting code & software for further information.

Data

Policy information about availability of data

All manuscripts must include a data availability statement. This statement should provide the following information, where applicable:

- Accession codes, unique identifiers, or web links for publicly available datasets
- A description of any restrictions on data availability
- For clinical datasets or third party data, please ensure that the statement adheres to our policy

Raw single cell and spatial transcriptomic datasets are deposited on Gene Expression Omnibus (accessions GSE295708 and GSE295862 respectively). Integrated single-nucleus and Xenium objects, together with auxiliary files, can be found at the Single Cell Portal (accessions SCP3116 and SCP3117 respectively). The following publicly available datasets were used in this study: human AT single nucleus transcriptomic data (Single Cell Portal, SCP1376 and GEO accession GSE176171); human reference genome (cf.10xgenomics.com/refdata-gex-GRCh38-2020-A.tar.gz); Molecular Signatures Database (MsigDB, https://www.gsea-msigdb.org/gsea/msigdb/); secreted proteins in the Human Protein Atlas (https://www.proteinatlas.org/humanproteome/tissue/secretome); motifs for SCENIC (resources.aertslab.org/cistarget/databases/homo_sapiens/hg38/refseq_r80/tc_v1/gene_based/); human GWAS (https://www.ebi.ac.uk/gwas/).

Research involving human participants, their data, or biological material

Policy information about studies with human participants or human data. See also policy information about sex, gender (identity/presentation), and sexual orientation and race, ethnicity and racism.

Reporting on sex and gender	Adult males and females were included in the study design. Primary and spatial cohort: N=Female 41, N=Male 16. Numbers or proportions of each sex are provided in the design figures, participant characteristics tables and methods. Sex specific analyses were not carried out because of the limited sample size, and insufficient power to detect and therefore report sex specific effects.
Reporting on race, ethnicity, or other socially relevant groupings	Ethnicity is reported for all study participants within participant meta-data files. Ethnicity is based on self reported NHS ethnicity categories, which were then grouped into one of: European White; South Asian; and Black, Black British, Caribbean or African. Confounding was controlled for by selecting obese cases and lean controls that were well matched for age, sex and ethnicity. These biological and other technical factors were also included as covariates in regression based analyses. Paired analyses were used to control for participant level factors before and after weight loss.
Population characteristics	Detailed population characteristics are provided in extended data table 1. Obese participants had BMI>35kg/m2, lean pariticipants BMI<25kg/m2. Groups were well matched for age (within 5yrs), sex and ethnicity. People with systemic illnesses not related to obesity were excluded, as were people with treated type 2 diabetes due to the potential effects of medications on adipose tissues.
Recruitment	Prospective participants were recurited sequentially from bariatric and other general surgery preassessment clinics. Study participants were then selected from the larger cohort to enable groups to be well matched for age sex and ethnicity. People with diabetes taking medication that might impact adipose tissue function were excluded. This may skew the obese study group towards less severe pathobiology but it is unlikely to impact results from the between group comparisions.
Ethics oversight	All participants gave informed consent. The study was approved by the London – City Road and Hampstead Research Ethics Committee, United Kingdom (reference 13/LO/0477). Human tissue validation also used samples from the Imperial College Healthcare Tissue Bank (ICHTB) – approved by Wales REC3 to release human material for research (reference 17/WA/0161).

Note that full information on the approval of the study protocol must also be provided in the manuscript.

Field-specific reporting

Please select the one below that is the best fit for your research. If you are not sure, read the appropriate sections before making your selection.

☒ Life sciences ☐ Behavioural & social sciences ☐ Ecological, evolutionary & environmental sciences

For a reference copy of the document with all sections, see nature.com/documents/nr-reporting-summary-flat.pdf

Life sciences study design

All studies must disclose on these points even when the disclosure is negative.

Sample size	Prospective sample sizes calculations were performed using the hierarchicell package and dispersion estimates from a pilot cohort of N=6 samples. Extreme trait (N=24 lean, N= 25 obese) and paired longitudinal (N=25 marked weight loss) sampling were combined with cell level analyses (>100K cells) to provide sufficient discovery power at the cell type and common/infrequent cell state levels.
Data exclusions	Two lean samples were excluded at integration because of very low cell numbers (technical failure) and very high lymphocyte counts (suggesting lymph node content in adipose tissue biopsy) respectively.
Replication	Each study participant/timepoint was considered an experimental replicate (N=24 lean, N=25 obese, N=25 weight loss). Individual samples were processed in pools (4-5 samples/pool; total of 6 pools/group). Sample pools for each experimental group were processed through to

sequencing in lean-obese-weight loss trios across 4 batches, to minimise between group batch effects. Single cell nucleus results were systematically replicated in independent samples (N=4/group) using a distinct spatial Xenium technology.

Randomization

Lean, obese and weight loss samples were processed in triplets, comprising the obese-weight loss pair and designated control, in random order to minimise batch effects.

Blinding

Unbiased genomic analyses were carried out unblinded. Blinding was not undertaken because single cell studies require iterative analysis, interpretaiton, and contextualization within the existing knowledge base. Tissue histological analyses were unblinded because manifest differences in human adipocyte sizes between conditions made blinding impossible. Cell culture validation experiments were quantified using unbiased imaging methods and did not thus require blinding.

Reporting for specific materials, systems and methods

We require information from authors about some types of materials, experimental systems and methods used in many studies. Here, indicate whether each material, system or method listed is relevant to your study. If you are not sure if a list item applies to your research, read the appropriate section before selecting a response.

Materials & experimental systems

n/a	Involved in the study	
☐	☒	Antibodies
☐	☒	Eukaryotic cell lines
☒	☐	Palaeontology and archaeology
☒	☐	Animals and other organisms
☒	☐	Clinical data
☒	☐	Dual use research of concern
☒	☐	Plants

Methods

n/a	Involved in the study	
☒	☐	ChIP-seq
☐	☒	Flow cytometry
☒	☐	MRI-based neuroimaging

Antibodies

Antibodies used

Immunohistochemistry:

anti-p21 Waf1/Cip1 (1:50, Cell Signalling, catalogue no. 2947, clone 12D1)
anti-rabbit IgG conjugated with polymeric horseradish peroxidase linker (25µg/ml, Leica Bond Polymer Refine Detection, DS9800).

Immunofluorescence:

anti-NAMPT (1:200, Affinity Biosciences #DF6059)
anti-TREM2 (clone D8I4C, 1:400, Cell Signalling #91068)
anti-TLR2 (clone TL2.1, 1:400, Invitrogen #14-9922-82)
anti-Stat3 (clone 124H6, 1:500, Cell Signalling # 9139S)
anti-c Jun (clone 60A8, 1:500, Cell Signalling # 9165S)
Goat anti-Rabbit Alexa Fluor 488 (1:200, Invitrogen #A-11034)
Donkey anti-Rabbit Alexa Fluor Plus 488 (1:250, Invitrogen # A32790)
Goat anti-Mouse Alexa Fluor Plus 647 (1:250, Invitrogen # A32728)

FACS
anti-human CD45 antibody conjugated to FITC (1:20, BioLegend # 304006, clone HI30)
anti-human CD9 antibody conjugated to APC-Fire (1:20, BioLegend # 312114, clone H19α)
anti-human FOLR2 antibody conjugated to APC (1:20, BioLegend # 391705, clone 94b/FOLR2)

Validation

Histology antibodies:

The anti-p21 Waf1/Cip1 (clone 12D1) antibody has been validated by Western blot analysis of p21 Waf1/Cip1 knockout HeLa cells. Its specificity on human tissue has been previously demonstrated by Zhu et al. (2019) using immunohistochemistry (IHC). In our study, we utilised a human breast cancer sample as a positive control to assess the specificity and functionality of the antibody dilution.

The anti-NAMPT (DF6059) antibody is specific to human, rat, and mouse tissues. According to the manufacturer, this antibody has been validated by IHC on rat adipose tissue and human esophageal cancer. Further specificity validation was conducted by Tang et al. (2020) using Western blot analysis with viral-induced NAMPT overexpression. In our study, we included a staining protocol using only the secondary antibody to evaluate potential non-specific secondary antibody binding in adipose tissue.

The anti-TREM2 (D8I4C) antibody has been validated by the manufacturer by western blot analysis of extracts from 293T cells transfected with a construct expressing Myc-tagged full-length human TREM2 protein or a mock construct.

The anti-TLR2 (TL2.1) has been validated by da Rocha et al. (2021) where its specificity its specificity to dectec TLR2 postive monocytes was shown.

The anti-Stat3 (124H6) antibody has been validated on several human cells lines by the manufacturer and this further confirmed by siRNA knock-down by Peng et al. (2017).

The anti-c Jun (60A8) antibody has valdated by the manufacturer by western blot analysis of extracts from control HeLa cells or c-Jun knockout HeLa cells and further confirmed by Yu et al. (2012) by knock-down using siRNA.

Zhu L, Ding R, Zhang J, Zhang J, Lin Z. Cyclin-dependent kinase 5 acts as a promising biomarker in clear cell Renal Cell Carcinoma. BMC Cancer. 2019 Jul 16;19(1):698. doi: 10.1186/s12885-019-5905-9. PMID: 31311512; PMCID: PMC6636025.

Tang JZ, Xu WQ, Wei FJ, Jiang YZ, Zheng XX. Role of Nampt overexpression in a rat model of Hashimoto's thyroiditis and its mechanism of action. Exp Ther Med. 2020 Apr;19(4):2895-2900. doi: 10.3892/etm.2020.8539. Epub 2020 Feb 21. PMID: 32256774; PMCID: PMC7086292.

da Rocha Sobrinho HM, Saar Gomes R, da Silva DJ, Quixabeira VBL, Joosten LAB, Ribeiro de Barros Cardoso C, Ribeiro-Dias F. Toll-like receptor 10 controls TLR2-induced cytokine production in monocytes from patients with Parkinson's disease. J Neurosci Res. 2021 Oct;99(10):2511-2524. doi: 10.1002/jnr.24916. Epub 2021 Jul 14. PMID: 34260774.

Peng C, Zhang S, Lei L, Zhang X, Jia X, Luo Z, Huang X, Kuang Y, Zeng W, Su J, Chen X. Epidermal CD147 expression plays a key role in IL-22-induced psoriatic dermatitis. Sci Rep. 2017 Mar 8;7:44172. doi: 10.1038/srep44172. PMID: 28272440; PMCID: PMC5341158.

Yu Z, Sato S, Trackman PC, Kirsch KH, Sonenshein GE. Blimp1 activation by AP-1 in human lung cancer cells promotes a migratory phenotype and is inhibited by the lysyl oxidase propeptide. PLoS One. 2012;7(3):e33287. doi: 10.1371/journal.pone.0033287. Epub 2012 Mar 15. PMID: 22438909; PMCID: PMC3305320.

Flow cytometry antibodies:

Antibodies for Flow cytometry were validated by the manufacturer (Biolegend) by staining of positive cells/tissue against matched Isotype control.

The anti-Human CD45-FITC (clone H130) was validated for FACS in cell lines by manufacturer and authors using human and murine cells. These include the validation of anti-Human CD45-FITC (clone H130) in FACS analysis of hepatic NK cells (Marquardt et al. 2015) and human hematopoietic progenitors (Chabi et al. 2019).

The anti-CD9-APC/Fire (clone H19a) antibody was validated for FACS using by Earley et al., (2021) in iPSC-derived neural cell mixture.

The anti-FOLR2 (clone 94b/FOLR2) has been validated by Western blot in analysis of folate receptor β (FRβ)–transfected B300-19 macrophage cells (Nagayoshi et al., 2005). Anti-human FRβ was validated in for FACS using human peripheral blood monocytes: https://www.biolegend.com/en-us/products/apc-anti-humanfolate-receptor-beta-fr-beta-antibody-15117

Chabi S, Uzan B, Naguibneva, I, Rucci, J, Fahy, L, Calvo, J, Arcangeli ML, Mazurier F, Pflumio F, Haddad R. Hypoxia Regulates Lymphoid Development of Human Hematopoietic Progenitors. Cell Rep. 2019 Nov 19;29(8):2307-2320.e6. doi: 10.1016/j.celrep.2019.10.050. PMID: 31747603

Marquardt N, Beziat V, Nystrom S, Hengst J, Ivarsson MA, Kekalainen E, Johansson H, Mjosberg, J, Westgren M, Lankisch TO, Wedemeyer, H, Ellis EC, Ljunggren HG, Michaelsson, J. Bjorkstrom NK. Cutting Edge: Identification and Characterization of Human Intrahepatic CD49a+ NK Cells. J Immunol (2015) 194 (6): 2467–2471. https://doi.org/10.4049/jimmunol.1402756

Earley AM, Burbulla LF, Krainc D, Awatramani R. Identification of ASCL1 as a determinant for human iPSC-derived dopaminergic neurons. Sci Rep. 2021 Nov 15;11(1):22257. doi: 10.1038/s41598-021-01366-4. PPMID: 34782629; PMCID: PMC8593045

Nagayoshi, R, Nagai, T, Matsushita, K, Sato, K. Sunahara, N, Matsuda, T, Nakamura, T, Komiya, S, Onda, M, Matsuyama, T. Effectiveness of anti–folate receptor β antibody conjugated with truncated Pseudomonas exotoxin in the targeting of rheumatoid arthritis synovial macrophages. Arthritis Rheum. 2005 Sep;52(9):2666-75. doi: 10.1002/art.21228. PMID: 16142741

Eukaryotic cell lines

Policy information about cell lines and Sex and Gender in Research

Cell line source(s)	Immortalized human adipose-derived stromal cells (Bmi-1/hTERT, iHASC) were acquired from Applied Biological Materials (T0540), derived from a female donor (30yrs-old).
Authentication	Cells were authentified by the manufactured by confirming the expression profile (CD44,CD73,CD105).
Mycoplasma contamination	Cell tested negative for mycoplasma at source.
Commonly misidentified lines (See ICLAC register)	No commonly misidentified cell lines used in the study.

Plants

Seed stocks

Report on the source of all seed stocks or other plant material used. If applicable, state the seed stock centre and catalogue number. If plant specimens were collected from the field, describe the collection location, date and sampling procedures.

Novel plant genotypes

Describe the methods by which all novel plant genotypes were produced. This includes those generated by transgenic approaches, gene editing, chemical/radiation-based mutagenesis and hybridization. For transgenic lines, describe the transformation method, the number of independent lines analyzed and the generation upon which experiments were performed. For gene-edited lines, describe the editor used, the endogenous sequence targeted for editing, the targeting guide RNA sequence (if applicable) and how the editor was applied.

Authentication

Describe any authentication procedures for each seed stock used or novel genotype generated. Describe any experiments used to assess the effect of a mutation and, where applicable, how potential secondary effects (e.g. second site T-DNA insertions, mosiacism, off-target gene editing) were examined.

Flow Cytometry

Plots

Confirm that:

☒ The axis labels state the marker and fluorochrome used (e.g. CD4-FITC).

☒ The axis scales are clearly visible. Include numbers along axes only for bottom left plot of group (a 'group' is an analysis of identical markers).

☒ All plots are contour plots with outliers or pseudocolor plots.

☒ A numerical value for number of cells or percentage (with statistics) is provided.

Methodology

Sample preparation

We used a modified SCENITH-based approach to evaluate human macrophage metabolic pathways ex vivo29 . Fresh subcutaneous AT was cut into ~2mm pieces (30ml HBSS (Gibco 14175-053) in a 50ml tube), washed and collected using a 100-µM cell strainer. Tissue was digested for 20-mins at 37C (3mg/ml Collagenase II (Sigma C6885) in methionine-free RPMI (Sigma R7513), 65 mg/L L-cystine dihydrochloride (Sigma C6727), 1x GlutaMAX (Gibco 35050061), 10% dialysed foetal calf serum (Gibco A3382001)). Digested tissue was filtered through a 100-µm strainer and digestion was terminated by addition of Methionine-free RPMI containing 10% FCS, followed by centrifugation (300-g at 4C for 7-min). Following resuspension in methionine-free RPMI (65 mg/L cystine, 10% FBS, 1x Glutamax), cells were plated (160-µl) into wells on a 96-well V-bottom plate. Cells were methionine starved for a further 15mins (total starvation ~45mins including digestion and isolation) before treatment with inhibitors or control media (40µl) for 15mins. The four treatments were media, 2-Deoxy-D-glucose (2-DG) (100mM final conc.; Sigma D8375), Oligomycin (2 µM final conc.; Sigma 495455) and 2-DG+Oligomycin (100mM & 2µM final conc. respectively). Homopropargylglycine (HPG; Cayman Chemical, 11785) was then added to wells at a final concentration of 500-µM and incubated for 30-min to initiate cell HPG uptake. An additional well received cells and media but no HPG and no treatment (click-chemistry negative control). After HPG uptake, cells were stained with zombie aqua live/dead stain (1:500 in PBS; BioLegend 423101) for 20-mins at RT in the dark, washed with PBS and then fixed with 2% PFA for 15-min.

Fixed cells were permeabilised (0.1% saponin/1% BSA in PBS) for 15-mins, washed with Click buffer (100mM Tris-HCL, pH7.4; Invitrogen 1556-027), and incubated with Fc receptor blocker (25µg/ml in PBS; Fc1, BD Biosciences 564765) for 10-mins. Cells were rewashed and incubated in 100µl of Click reaction mix in the dark at RT for 30-mins. Click reaction mix was made sequentially, adding CuSO4 (Final conc. 0.5mM; Sigma 209198), THPTA (Final conc. 2mM; Antibodies.com A270328), Sodium Ascorbate (Final conc., 10mM; Sigma, A7631) and then AZDye 555 (Final conc. 25µM; Vector Laboratories, CCT1479) to Click buffer (final concentration, 100mM Tris-HCL).

After Click chemistry exposure, cells were washed using FACS buffer (PBS, 1% BSA, 5mM EDTA, 25mM HEPES), and stained with antibody mix (FACS buffer, anti-CD45 FITC [1:20; H130, Biolegend 304006], anti-FOLR2 APC [1:20; 94b/FOLR2, Biolegend 391705], anti-CD9 APC-fire [1:20; H19α, Biolegend 312114], Fc Block reagent [25µg/ml]) at 4C in the dark for 30mins. After re-washing, cells were filtered (35µM cap strainer) for FACS analysis

Instrument

Sony ID7000

Software

AF Finder tool , Flowjo

Cell population abundance

CD45 positive immune cells, median 9.9% (IQR 6.5-12.3%) of live single cells. FORL2 positive TRMs, median 24.4% (IQR17.7-28.8%) of CD45 positive immune cells. CD9 positive LAMs, median 9.9% (IQR 7.7-11.9%) of CD45 positive immune cells.

Gating strategy

LAM and TRM gating for SCENITH-based bioenergetic studies. Gate 1: All cells (FSC-A and SSC-A). Gate 2: Single cells (FSC-A and FSC-H). Gate 3: Live immune cells (CD45-hi, Zombie-Aqua-lo). Gate 4A: TRM cells (FORL2-hi, CD9-lo). Gate 4B: LAM cells (FORL2-lo, CD9-hi). AZ555: Click Chemistry histogram of AZ555 for respective Gates 4A and 4B. Fluorochrome and autofluorescence signatures were identified in unstained aliquots of each sample using the "AF Finder" software feature, were used to unmix the signals in fully stained samples with the built-in WLSM algorithm.

☒ Tick this box to confirm that a figure exemplifying the gating strategy is provided in the Supplementary Information.
