# Supplementary material for: Selective remodelling of the adipose niche in obesity and weight loss
Source: Nature. 2025 Jul 9;644(8077):769–79. doi: 10.1038/s41586-025-09233-2 (PMC12367556; doi:10.1038/s41586-025-09233-2)
Supplement: Supplementary file 4 — Supplementary Tables 1-12 [file 41586_2025_9233_MOESM4_ESM.zip › 2024-05-10393C-s4/SupplementaryTableLegends.docx]

**Supplementary Table 1**

Cell Type marker table, obtained from running Wilcoxon test on the single-nucleus global object. Contains log2 fold-change, p-value, percentage expression and mean expression of each cell type vs everything else.

**Supplementary Table 2**

Cell State marker table, obtained from running Wilcoxon test within each cell type specific single-nucleus object. Contains log2 fold-change, p-value, percentage expression and mean expression of each cell state vs everything else within the respective cell type.

**Supplementary Table 3**

Cell Type marker table, obtained from running Wilcoxon test on the Xenium global object. Contains log2 fold-change, p-value, percentage expression and mean expression of each cell type vs everything else.

**Supplementary Table 4**

Cell State marker table, obtained from running Wilcoxon within each cell type specific Xenium object. Contains log2 fold-change, p-value, percentage expression and mean expression of each cell state vs everything else within the respective cell type.

**Supplementary Table 5**

Differential expression of LAM ST2 vs LAM ST1 in the single-nucleus dataset, obtained with Wilcoxon test. Contains log2 fold-change, p-value, percentage expression and mean expression of each.

**Supplementary Table 6**

Statistics of all COMPASS reactions in each cell type, for Lean vs Obese and Weight Loss vs Obese. Contains reaction information, p-value (Wilcoxon test), adjusted p-value and Cohen’s D.

**Supplementary Table 7**

All differential expression in the single-nucleus dataset using NEBULA for Obese vs Lean and Weight Loss vs Lean, per Cell Type. Contains p-value, FDR/Bonferroni p-value (for WL and LN comparisons respectively), standard error (se), log2 fold change and percentage of expressing nuclei.

**Supplementary Table 8**

Differential expression in the Xenium dataset using Wilcoxon test for Obese vs Lean and Obese vs Weight Loss, per cell type. Contains log2 fold-change, p-value, FDR correction, percentage expression and mean expression per comparison.

**Supplementary Table 9**

Differential pySCENIC regulons for each cell state vs all other cell states, within each cell type, using Wilcoxon test. Contains log2 fold change and p-value.

**Supplementary Table 10**

Differential pySCENIC regulons for Obese vs Lean and Obese vs Weight Loss, per cell type, using Wilcoxon test. Contains log2 fold change, average regulon score and p-value per comparison

**Supplementary Table 11**

Differential pySCENIC regulons limited to metabolic genes, using Wilcoxon test for Lean vs Obese and Weight Loss vs Obese Loss. Contains log2 fold change and p-value per condition

**Supplementary Table 12**

Differential expression in the single-nucleus dataset, for each stressed cell state vs respective basal state, using Wilcoxon test. Contains log2 fold-change, p-value, FDR correction, percentage expression and mean expression per comparison and a flag to indicate if the gene is present in the Xenium panel.
